# Whole-Genome Resequencing Identifies SNPs in Sucrose Synthase and Sugar Transporter Genes Associated with Sweetness in Coconut

**DOI:** 10.3390/plants13182548

**Published:** 2024-09-11

**Authors:** Manlika Khongmaluan, Wanchana Aesomnuk, Reajina Dumhai, Mutiara K. Pitaloka, Yong Xiao, Rui Xia, Tippaya Kraithong, Natthaporn Phonsatta, Atikorn Panya, Vinitchan Ruanjaichon, Samart Wanchana, Siwaret Arikit

**Affiliations:** 1 Department of Agronomy, Faculty of Agriculture at Kamphaeng Saen, Kasetsart University Kamphaeng Saen Campus, Nakhon Pathom 73140, Thailand; manlika.kong@ku.th; 2 Rice Science Center, Kasetsart University, Nakhon Pathom 73140, Thailand; waesomnuk@gmail.com (W.A.); reajina.d06@gmail.com (R.D.); 3 Research Center for Applied Botany, National Research and Innovation Agency, Jl. Raya Jakarta-Bogor KM 46, Bogor 16911, Indonesia; mutiarakp@gmail.com; 4 Coconut Research Institute, Chinese Academy of Tropical Agriculture Sciences, Haikou 571339, China; xiaoyong_coconut@163.com; 5 State Key Laboratory for Conservation and Utilization of Subtropical Agro-Bioresources, College of Horticulture, South China Agricultural University, Guangzhou 510640, China; rxia@scau.edu.cn; 6 Chumphon Horticulture Research Center, Department of Agriculture, Bangkok 10900, Thailand; tkraitong_28@hotmail.com; 7 National Center for Genetic Engineering and Biotechnology (BIOTEC), 113 Thailand Science Park, Pahonyothin Road, Khlong Luang, Pathum Thani 12120, Thailand; natthaporn.pho@biotec.or.th (N.P.); atikorn.pan@biotec.or.th (A.P.); vinitchan.rua@biotec.or.th (V.R.); samart.wan@biotec.or.th (S.W.)

**Keywords:** coconut, sweetness, sucrose metabolism, sucrose synthase, sugar transporter, allele mining, whole-genome resequencing

## Abstract

Coconut (*Cocos nucifera* L.) is an important agricultural commodity with substantial economic and nutritional value, widely used for various products, including coconut water. The sweetness is an important quality trait of coconut water, which is influenced by genetic and environmental factors. In this study, we utilized next-generation sequencing to identify genetic variations in the coconut genome associated with the sweetness of coconut water. Whole-genome resequencing of 49 coconut accessions, including diverse germplasm and an F_2_ population of 81 individuals, revealed ~27 M SNPs and ~1.5 M InDels. Sugar content measured by °Bx was highly variable across all accessions tested, with dwarf varieties generally sweeter. A comprehensive analysis of the sugar profiles revealed that sucrose was the major sugar contributing to sweetness. Allele mining of the 148 genes involved in sugar metabolism and transport and genotype–phenotype association tests revealed two significant SNPs in the hexose carrier protein (Cnu01G018720) and sucrose synthase (Cnu09G011120) genes associated with the higher sugar content in both the germplasm and F_2_ populations. This research provides valuable insights into the genetic basis of coconut sweetness and offers molecular markers for breeding programs aimed at improving coconut water quality. The identified variants can improve the selection process in breeding high-quality sweet coconut varieties and thus support the economic sustainability of coconut cultivation.

## 1. Introduction

Coconut (*Cocos nucifera* L.) is widely known for its many uses, especially as a food and energy crop. The fruit is the most valuable part of the coconut and provides a range of products such as coconut water, virgin coconut oil, copra and coconut milk [1]. Coconut cultivation covers more than 12 million hectares in 92 countries, underlining its global agricultural importance [2]. The versatility and economic importance of the fruit make it indispensable for both local consumption and international trade [3]. The world’s coconut populations are divided into two main groups: the Indo-Atlantic group and the Pacific group, which includes subgroups such as Southeast Asia, Micronesia, Polynesia, Melanesia and the Pacific coast of South and Central America [4,5]. Coconut varieties are divided into tall and dwarf tree varieties [6]. Tall coconuts, which cross-pollinate, have greater genetic diversity than dwarf coconuts, which self-pollinate and have less genetic diversity [5,7]. This genetic diversity is crucial for breeding programs and the development of new varieties with desirable traits. Dwarf varieties can be further subdivided based on the color of the nuts, e.g., green, yellow or brown [8]. Tall coconuts, which are more common worldwide, are valued primarily for their mature fruits, while dwarf coconuts are valued for the coconut water and tender flesh of their young fruits [5]. The liquid endosperm, which consists predominantly of glucose, sucrose and fructose, is richer in dwarf varieties, making them ideal for the production of coconut water [9]. Popular varieties for coconut water consumption are Green Dwarf, King Coconut, Aromatic Green Dwarf (Nam Hom) and Chowgat Orange Dwarf [9]. Coconut water contains sugars, minerals, vitamins, amino acids, enzymes, volatile aromatic compounds and other biochemical compounds [10]. Studies on the physicochemical properties of different coconut varieties have shown that dwarf coconuts, with their higher pH and lower titratable acidity, have a better flavor profile characterized by sweetness and acidity [10,11]. Sweetness is a crucial quality attribute that consumers value in coconut water. Improving the sweetness of coconuts is important for increasing their market value. The sweetness of coconut water is influenced by both the genetic makeup of the coconut and environmental factors such as soil type and climate [12]. In addition, it depends on the stage of maturity, with young coconuts generally producing sweeter water. Coconuts usually take 12 months to fully mature after pollination, with the sugar content in coconut water peaking after about 7 to 8 months before gradually decreasing [12]. Therefore, most coconut farms harvest the fruit at this optimum age to ensure the best quality of coconut water. Understanding the genetic and metabolic determinants of coconut sweetness has practical implications for breeding programs to improve this trait.

The sugar metabolism process is principally regulated by four important enzymes: neutral invertase, acid invertase, sucrose phosphate synthase and sucrose synthase [13,14]. Understanding the genetic basis of these metabolic pathways is crucial for the development of breeding strategies that can improve the sweetness of coconut water. Plants produce sucrose in photosynthetically active tissue through the process of photosynthesis and transport it into the phloem. Sucrose is then moved to sink organs, where it can be rapidly hydrolyzed into glucose and fructose [13,15]. Research on other crops has shown that manipulating the expression of genes involved in sugar metabolism can significantly alter the sugar content of fruits [16]. In tomato fruits, pathways involving invertase and sucrose synthase play an important role in sucrose metabolism [17]. In maize, sweetness is primarily controlled by the *shrunken*-*2* (*sh2*) gene, which affects sucrose synthase, resulting in higher sugar accumulation in the kernels. The manipulation of these genes has helped to develop super sweet maize varieties that have a higher sugar content compared with normal maize [16]. Although research on the genes encoding the essential enzymes involved in sugar production has been conducted in a number of plants [14,16], no comparable study has been conducted on coconuts. In this study, we utilized the next-generation sequencing (NGS) technology of a collection of coconut differing in sweetness levels to characterize the genes involved in the sugar biosynthetic pathway in coconut and identify variants associated with sweetness. By analyzing these genes, we successfully identified natural variants in two candidate genes associated with the sweetness in coconut. The results of our study provide a fundamental understanding of genetic control over coconut sweetness and offer a pathway for genetic improvement of coconut cultivars through targeted breeding strategies.

## 2. Results

### 2.1. Whole-Genome Resequencing, Variant Calling and Population Study of Coconut Populations

The whole genome of 2 coconut populations, a germplasm of 49 coconut accessions and an F_2_ population of 81 individuals, was sequenced. The 49 coconut accessions represented a diversity of coconuts in Thailand, including 17 dwarf and 32 tall coconuts (Table 1). The F_2_ population was derived from a cross between a dwarf coconut and a tall coconut (Table S1). Whole-genome sequencing of the 130 coconut samples yielded a total of 42,044 million 150 bp paired-end reads with an average depth of 14.16× and an average genome coverage of 98.71%. After mapping with an in-house coconut reference genome, a set of 27,000,601 common SNPs was identified, along with 1,540,742 InDels (Table 1 and Table S1).

**Figure 1:**
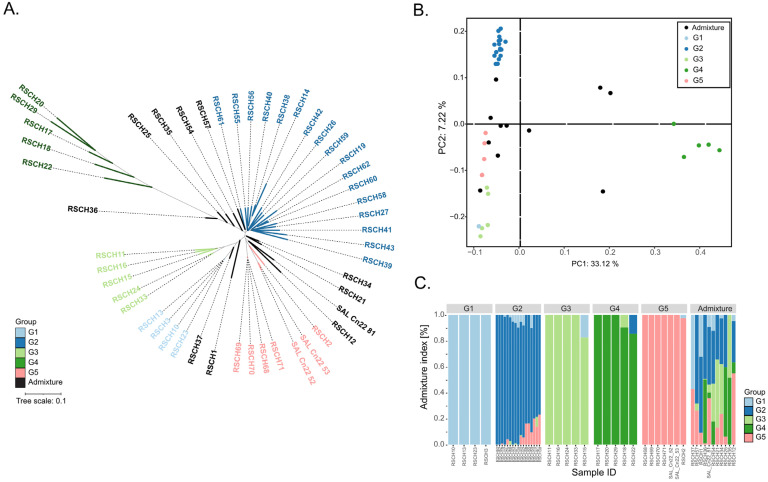
Population study of the 49 coconut accessions. (**A**) UPGMA phylogenetic tree. (**B**) Principal coordinate analysis (PCoA). (**C**) STRUCTURE analysis. Different genetic groups are highlighted by different colors.

To investigate the population structure and genetic diversity of the 49 coconut accessions, we performed three analyses, namely phylogenetic tree analysis, principal coordinate analysis (PCoA) and STRUCTURE analysis. The genotype data used for these analyzes were 18,379,163 filtered SNPs of the 49 coconut accessions. The genetic relationships analyzed by UPGMA-based clustering and the population structure analyzed by PCoA and STRUCTURE revealed 5 major groups (G1–G5) among the 49 coconut accessions: G1, consisting of 4 dwarf accessions; G2, the largest group, consisting of 17 tall accessions; G3, consisting of 5 colored dwarf accessions; G4, consisting of 5 tall accessions with the highest distance matrix to the other groups; G5, consisting of 7 dwarf accessions (Figure 1; Table 1). Based on the results of the STRUCTURE analysis, 11 accessions were considered as admixtures. The population study results confirmed the diversity of coconut accessions used in this study.

### 2.2. Analysis of Sweetness and Sugar Profile

**Figure 2:**
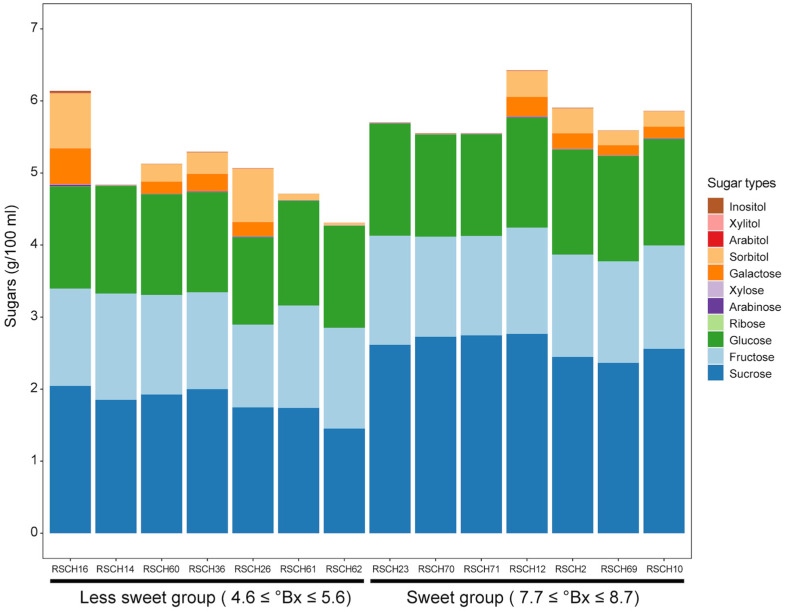
The sugar content in coconut water from accessions with varying sweetness levels. A stacked bar graph illustrates the quantities of different sugars and sugar alcohols measured in coconut water extracted from 7-month-old fruits.

The sweetness of the coconut water was derived from the sugar content (°Bx) measured with a refractometer. The evaluated sugar content of the 49 coconut accessions ranged from 4.60 to 8.70 °Bx, with an average of 6.66 °Bx (Table 1). The sugar content of the dwarf varieties averaged 7.32 °Bx and that of the tall varieties 6.31 °Bx. The sweetest coconut water was found in the dwarf accession RSCH23 (8.7 °Bx) and the least sweet coconut water in the tall accession RSCH62 (4.6 °Bx) (Table 1). The evaluated sugar content of the 81 F_2_ individuals ranged from 5.40 to 7.60 °Bx, with an average of 6.46 °Bx. We then selected seven representatives of the coconut accessions with the highest sugar content values (namely the sweet group) of 7.7 ≤ °Bx ≤ 8.7 and seven accessions with the lowest sugar content values (namely the less-sweet group) of 4.7 ≤ °Bx ≤ 5.6 to analyze the sugar profile, focusing on disaccharides, i.e., sucrose; monosaccharides, i.e., fructose, glucose, ribose, arabinose, xylose and galactose; and sugar alcohols, i.e., sorbitol, arabitol, xylitol and inositol with HPAEC-PAD. Of these, the content of fructose, glucose and sucrose was higher in all the accessions studied (Figure 2). The type of sugar that was present in the greatest quantity in all accessions was sucrose. When comparing the two groups of coconut accessions, the average sucrose content in the coconut water of the accessions was significantly higher in the sweet group (2.60 ± 0.15 g/100 mL) than in the less-sweet group (1.82 ± 0.20 g/100 mL). For the other sugars and the sugar alcohol, there was no clear trend when comparing the two groups. This indicates that the accumulation of sucrose content is probably related to the higher sweetness of the coconut water.

### 2.3. Identification of Genes Associated with Sweetness in Coconut

We used a gene mining approach to identify candidate genes associated with the sweetness of coconut water. A total of 148 genes involved in sugar metabolism and sugar transport were identified based on gene functional annotations in the coconut genome. These included genes encoding sucrose synthases and sugar transporters (Table S2). We then identified variants, i.e., SNPs and InDels, present in each gene. A total of 14,471 variants were identified with high confidence and compared among the 49 coconut accessions. Of these, the 871 variants were annotated with an effect on gene translation, i.e., missense variants causing amino acid changes and those causing stop-loss, stop-gain and frame shifts. Subsequently, these functional variants were selected to perform genotype–phenotype association tests with the sugar content in the °Bx scale determined in the 49 coconut accessions and an F_2_ population of 81 plants (Table S3). As a result, among the functional SNP and InDel variants, 57 SNPs within 35 genes were found to be significantly associated with sweetness in the 49 coconut accessions (*p* < 0.01; Table 2). After validation in the F_2_ population segregating for sugar content (°Bx), only SNPs 1_157345938 (A/G) and 9_119160271 (C/A) were found to be significantly associated with the trait (*p* < 0.05).

**Figure 3:**
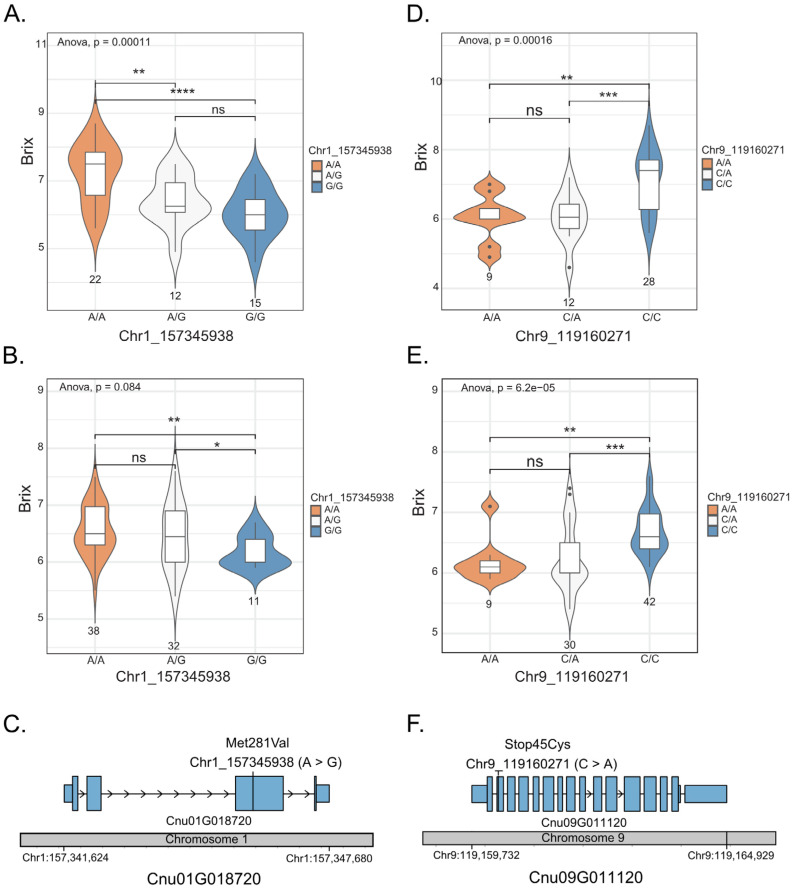
Genotype–phenotype association on two genes, Cnu01G018720 and Cnu09G011120. The box plot/violin plots display the three different genotypes at SNP 1_157345938 on the gene Cnu01G018720, analyzed in 49 accessions (**A**) and 81 F_2_ individuals (**B**). The box plot/violin plots display the three different genotypes at SNP 9_119160271 on the gene Cnu09G011120, analyzed in 49 accessions (**D**) and 81 F_2_ individuals (**E**). The medians are indicated by solid horizontal lines in the box plots. The structure of Cnu01G018720 (**C**) and Cnu09G011120 (**F**) shows UTRs (small blue boxes), exons (large blue boxes) and introns (arrow lines). The grey bar represents the chromosome, with SNP positions in each gene indicated by vertical bars. * *p* < 0.05; ** *p* < 0.01; *** *p* < 0.001; **** *p* < 0.0001; ns (not significant).

Among the 49 coconut accessions, the average °Bx value of the accessions with the homozygous genotype (A/A) at position 1_163346328 was 7.23 °Bx, which was significantly higher than that of the accessions with the homozygous genotype (GG) at the same position, which was 6.03 °Bx (Figure 3A). The average °Bx value of the accessions with the heterozygous genotype (A/G) was not significantly different from that of the accessions with the homozygous genotype (G/G) but it was significantly different from that of the accessions with the homozygous genotype (A/A). For SNP position 9_119160271, the average °Bx value of the accessions with the homozygous genotype (A/A; 7.11 °Bx) was also significantly higher than that of the accessions with the homozygous genotype (C/C; 6.04 °Bx). The average °Bx value of the accessions with the heterozygous genotype (A/C) was not significantly different from that of the accessions with the homozygous genotype (A/A) but it was significantly different from that of the accessions with the homozygous genotype (C/C; Figure 3D). A similar trend was confirmed in the F_2_ population (n = 81; Figure 3B,E). Based on these results, we considered the two SNPs as the candidate SNPs associated with coconut sweetness. The SNP 1_157345938 (A/G) was identified in exon 3 of the gene Cnu01G018720 (hexose carrier protein), where the nucleotide change A > G should have an effect on an amino acid change Met281Val (Figure 3C). SNP 9:119160271 was identified in exon 2 of the gene Cnu09G011120 (sucrose synthase 7), in which the nucleotide change C > A had an effect on a stop codon gain (TGC > TGA; Figure 3F).

## 3. Discussion

Coconut water is one of the best-known natural refreshing drinks. It is known for its health-promoting and energizing properties due to its sugars, dietary fiber, antioxidants, vitamins, minerals and phytohormones [10,18]. Over time, coconut water has gained popularity as a beverage that possesses both functional and nutritional properties, which has contributed to its economic growth. Improving the quality of coconut water will therefore increase its market value. The quality of coconut water is mainly related to its sweetness, which is mainly determined by its sugar content. The present study focused on identifying the genetic basis associated with the sweetness of coconut water so that the results can be used in a molecular breeding program to improve coconut for high quality coconut water. The coconut populations used in this study were a diverse germplasm of coconut in Thailand, which included 49 accessions of tall and dwarf types of Thai and international coconut cultivars and an F_2_ population of 81 individuals. The coconut water from the young fruits (7-month-old) of these coconut accessions and lines had different degrees of sweetness, as determined by the soluble solids’ content in degrees Brix (°Bx). The coconut water of most dwarf coconut accessions was sweeter than that of the coconut accessions of the tall group. This was confirmed by a recent study on the physicochemical properties and sensory acceptability of different coconut varieties, which found that the water of the dwarf coconut was more acceptable and palatable than the water of the tall coconut [11]. The comprehensive analysis of sugar profiles, including sugar alcohols, in coconut water provides insights into the broader metabolic pathways active in the different coconut genotypes. The results of the HPAEC-PAD analysis revealed the variations in the major sugar components such as sucrose, glucose and fructose, which collectively define the sweetness of coconut water. Our results show significant differences in sucrose concentration between the sweet and less-sweet coconut groups, suggesting that the different sweetness of each coconut group is due to their own sucrose metabolism. Higher sucrose concentrations in sweeter varieties indicate more efficient sucrose synthesis or a lower rate of sucrose degradation.

NGS technologies can be used to analyze diverse germplasm, leading to the discovery of candidate genes and trait-specific diagnostic markers. Rapid advances in NGS technologies have significantly reduced the cost and increased the speed of whole genome sequencing (WGS). This makes it possible to scan whole genomes or specific pathways to identify candidate genes in different accessions. For example, the application of NGS in tree breeding has led to the identification of candidate genes for traits such as fruit quality [19]. The application of WGS in this study provided a comprehensive overview of the genetic landscape of coconut cultivars. The groups of coconut accessions occurring in Thailand classified by the WGS-derived SNPs were similar to those identified by the GBS-derived SSRs in our previous study [6], except that the dwarf coconut cluster was further divided into three subgroups (G1, G3 and G5) in this study. This could be due to the high resolution of SNP markers used in this study compared with the much lower number of SSR markers used in the previous study. The genome-wide variants (SNPs and InDels) obtained from the WGS data were used for allele mining to identify candidate genes associated with sweetness in the 49 different coconut accessions in this study. Allele mining with NGS is a cost-effective method for identifying genetic variation in candidate genes and loci that are important for crop improvement. For instance, the allelic mining has been used to explore allelic diversity for nutrient-rich traits in rice [20]. The results of genotype-phenotype association analysis conducted on the 49 coconut accessions addressed two SNPs within a hexose carrier protein and a sucrose synthase gene. These SNPs were found to be strongly associated with variations in coconut sweetness, highlighting the potential of marker–trait association studies in linking genetic variants to the trait. Sucrose synthase plays a crucial role in sugar metabolism by catalyzing the reversible conversion of sucrose and uridine diphosphate (UDP) into UDP-glucose and fructose [21,22]. This enzyme plays a dual role in the synthesis and degradation of sucrose in response to metabolic demands, which is consistent with the observed differences in sugar content between different coconut genotypes [22]. This enzyme not only contributes to sucrose metabolism, but also provides UDP-glucose for the synthesis of cellulose in the cell wall, highlighting its role in structural and metabolic processes within the plant [23]. The significant SNP (C > A) in the *sucrose synthase* (Cnu09G011120) was annotated to possess the stop-gain effect that leads to the prematurely stop codon present in the exon 2, suggesting that this change may be critical for the increase in sucrose content of coconut water. Likewise, the hexose carrier protein (Cnu01G018720) may also affect the sugar metabolism in the coconut water. Hexose carrier proteins, also known as hexose transporters or sugar transporters, are integral to various physiological processes related to sugar metabolism [24]. The SNP (A > G) in this gene leads to the amino acid change (Met281Val) that may alter the activity of the enzyme and thereby directly modulate sucrose content. However, further studies are still needed to confirm the function of these two genes in sucrose metabolism in coconut. Nevertheless, the results from our study are crucial for the development of molecular markers that can be used in marker-assisted selection (MAS) to breed coconut varieties with improved sweetness that meet market demands and support the economic sustainability of coconut cultivation. In summary, this research provides a fundamental understanding of genetic control over coconut sweetness and offers a pathway for genetic improvement of coconut cultivars through targeted breeding strategies. The integration of genomic tools with traditional breeding approaches promises to improve the commercial viability and attractiveness of coconut products and support the sustainable development of the coconut industry.

## 4. Materials and Methods

### 4.1. Plant Materials

The coconut population used for this population study and allele mining consisted of 49 coconut accessions, including 32 accessions of tall coconut and 17 accessions of dwarf coconut. A total of 81 F_2_ plants from the cross of a tall coconut and an aromatic coconut were also used to validate the candidate SNPs. The 49 coconut accessions were grown at the KU-BEDO Coconut Biobank, Kasetsart University, Kamphaeng Saen Campus, Nakhon Pathom, Thailand. The F_2_ population was grown at the Trang Horticulture Research Center, Trang, Thailand. The plants were irrigated daily and fertilized every three months with chicken manure as organic fertilizer. Only one plant per accession was used to perform the experiment.

### 4.2. DNA Extraction, Whole-Genome Sequencing and Variant Calling

Young fresh coconut leaves were collected for DNA extraction. High-quality DNA from coconut leaf samples was extracted using the DNeasy Plant Mini Kit (Qiagen, Hilden, Germany). Whole genome sequencing (WGS) was performed using MGI-seq technology. The WGS process was performed by China National GeneBank (CNGB), Shenzhen, China. The sequence data obtained from whole genome sequencing of 49 coconut accessions and 81 F_2_ individuals were analyzed to identify variants, SNPs and InDels across the genome using the GATK software suite (4.6.0.0) [25]. The reference genome used for read mapping and variant calling was an unpublished reference genome of an aromatic dwarf coconut.

### 4.3. Population Structure Analysis

The genetic distance among the 49 coconut accessions was determined using Nei’s standard dissimilarity distance, based on 18,379,163 filtered SNPs, and a UPGMA phylogenetic tree was constructed with 500 bootstraps in MEGA X [26]. STRUCTURE analysis utilized a Bayesian model-based clustering algorithm in STRUCTURE version 2.3.4 [27], applying the admixture model with correlated allele frequencies. Each genetic cluster (K) value (K = 1–10) was analyzed in three independent runs, with a burn-in of 100,000 iterations followed by 100,000 Markov chain Monte Carlo (MCMC) repetitions. LnP(D) values were plotted to identify the ∆K plateau [28]. Genotypes were assigned to subpopulations if their probability of membership (Q value) was 0.70 or higher, while accessions with Q < 0.70 were considered genetically admixed. Principal coordinate analysis (PCoA) was conducted using DARwin 6.0.21 [29], and the final results were visualized with the ggplot2 R package [30].

### 4.4. Sweetness Evaluation and Sugar Profile Analysis

For the evaluation of sweetness, a total of five fruits (7-month-old) were collected from each coconut accession. The coconut water from each fruit was then collected and the total soluble sugar content was measured using a digital handheld reflectometer (MyBrix, Mettler-Toledo (Thailand) Co., Ltd., Bangkok, Thailand). The sweetness was expressed in degrees Brix (°Bx). Analysis of the different sugars from the 14 coconut accessions representing the sweet group and the less-sweet group was performed using high performance anion exchange chromatography with pulsed amperometric detector (HPAEC-PAD), model Dionex ICS-6000 DC and Chromeleon Console software version 7.2.10 (Thermo Fisher Scientific, Waltham, MA, USA). A CarboPac PA1 analytical column (4 × 250 mm) and a CarboPac PA1 guard column (4 × 50 mm) were used together with standard substances for quantification. Each accession was analyzed four times.

### 4.5. Allele Mining Genes in Sugar Metabolism and Transport and Genotype–Phenotype Association Analysis

The genes involved in sugar metabolism and sugar transport in coconuts were collected based on the gene functional annotations. The SNPs and InDels present in each of these genes were determined and compared among the 49 coconut accessions and 81 F_2_ individuals. The effects of SNPs and InDels in each gene were estimated using SnpEff [31]. A genotype–phenotype association analysis was performed on the 49 coconut accessions and 81 F_2_ individuals with a simple regression method using the lm() function in R (http://www.r-project.org, accessed on 1 June 2024). The genotype data used for the analysis were the SNPs and InDels in the genes and the phenotype data were the sugar content measured in degrees Brix (°Bx).

## 5. Conclusions

Our results provide valuable insights into the genetic control of coconut sweetness. The two significant SNPs identified in the sucrose synthase and hexose carrier genes could be used in breeding programs to develop better coconut varieties with improved sweet tastes, ultimately meeting consumer demands and supporting the economic viability of coconut breeding. This research not only contributes to our understanding of coconut biology but also has practical implications for the coconut industry, where there is a growing demand for high-quality sweet coconut water.

## Figures and Tables

**Table 1:** List of coconut accessions and details on genetic groups, sweetness (Brix) and sequencing and variant statistics.

Accession ID	Name	Group	Type	Brix	Raw Read (Million)	Mapped Reads (%)	Average Depth	No. of SNPs	No. of InDels
RSCH1	Mu Si Luang	Admixture	Tall	6.8	360.14	98.96	15.85	620,252	59,543
RSCH2	Nam Wan	G5	Dwarf	8.0	361.69	98.96	15.90	770,210	72,936
RSCH3	Pathiw	G1	Dwarf	6.0	350.02	98.85	15.40	605,095	59,835
RSCH10	Tung Kret	G1	Dwarf	7.7	342.66	98.89	15.05	600,709	58,792
RSCH11	MuSi Som (orange)	G3	Dwarf	7.5	349.96	98.74	15.36	646,291	61,172
RSCH12	Puang Roi	Admixture	Tall	8.1	290.36	98.92	12.80	673,944	59,142
RSCH13	Nam Hom #1	G1	Dwarf	7.5	350.05	98.94	15.41	607,824	59,930
RSCH14	Pak Chok #1	G2	Tall	5.6	349.98	98.88	15.38	1,293,375	117,102
RSCH15	Papua Nugini brown dwarf	G3	Dwarf	7.4	364.15	98.97	16.03	636,506	60,933
RSCH16	Cameroon yellow dwarf	G3	Dwarf	5.6	309.68	98.80	13.64	615,516	59,726
RSCH17	West African Tall #1	G4	Tall	6.0	350.02	98.96	15.38	5,078,243	280,368
RSCH18	West African Tall #2	G4	Tall	6.2	321.10	98.72	14.05	4,091,019	228,083
RSCH19	Kalok #1	G2	Tall	6.0	306.81	98.77	13.43	3,817,502	227,197
RSCH20	Talai Roi	G4	Tall	6.8	323.94	98.79	14.24	3,075,937	180,670
RSCH21	Tahiti	Admixture	Tall	7.1	330.79	98.76	14.46	4,704,771	270,072
RSCH22	Pak Chok #2	G4	Tall	7.0	322.17	98.73	14.13	4,435,916	251,018
RSCH23	Nok Khum	G1	Dwarf	8.7	355.14	98.85	15.61	613,071	60,224
RSCH24	Nalike (yellow)	G3	Dwarf	6.2	366.09	98.82	16.11	644,087	62,993
RSCH25	Chumphon 60	Admixture	Tall	6.0	344.14	97.05	14.75	9,243,464	469,432
RSCH26	Thai Tall #6	G2	Tall	5.2	307.77	98.59	13.42	3,881,637	216,543
RSCH27	Tapsakae	G2	Tall	5.9	371.28	98.62	16.21	4,695,620	253,492
RSCH29	West African Tall #3	G4	Tall	6.3	353.19	98.57	15.48	3,595,949	206,013
RSCH33	Malayan yellow dwarf	G3	Dwarf	7.2	378.57	98.31	16.56	617,018	60,060
RSCH34	Chumphon 2	Admixture	Tall	6.2	325.48	98.74	14.28	4,229,504	241,368
RSCH35	MaWa	Admixture	Tall	6.0	321.83	98.56	14.05	9,608,775	482,085
RSCH36	Khom #1	Admixture	Dwarf	5.5	304.23	98.38	13.27	8,237,781	414,489
RSCH37	Maphraw Fai	Admixture	Tall	6.5	378.46	98.56	16.60	619,315	60,671
RSCH38	Pak Chok #3	G2	Tall	6.2	330.42	98.50	14.46	3,587,634	222,083
RSCH39	Pak Chok #4	G2	Tall	6.3	268.22	98.56	11.78	2,428,643	144,064
RSCH40	Pak Chok #5	G2	Tall	6.9	283.06	98.48	12.39	3,694,255	215,166
RSCH41	Pleuk Wan	G2	Tall	7.2	334.88	98.64	14.67	3,943,537	234,280
RSCH42	Saw	G2	Tall	7.4	299.19	98.62	13.08	4,195,785	232,817
RSCH43	Khom #2	G2	Tall	6.8	356.35	98.61	15.63	2,418,467	148,546
RSCH54	Thai Tall #1	Admixture	Tall	5.7	324.68	98.75	14.23	5,883,454	314,838
RSCH55	Thai Tall #2	G2	Tall	6.1	323.13	98.65	14.16	4,201,893	250,662
RSCH56	Thai Tall #3	G2	Tall	5.8	356.15	98.58	15.60	4,116,409	249,872
RSCH57	Thai Tall #4	Admixture	Tall	6.4	349.92	98.70	15.33	4,026,836	224,703
RSCH58	Thai Tall #4	G2	Tall	6.4	325.44	98.69	14.24	4,316,205	232,021
RSCH59	Thai Tall #5	G2	Tall	6.5	334.18	98.69	14.63	3,917,338	223,471
RSCH60	Kalok #2	G2	Tall	5.5	352.21	98.75	15.43	4,189,965	242,214
RSCH61	Kalok #3	G2	Tall	4.9	324.38	98.80	14.23	4,053,617	234,133
RSCH62	Kalok #4	G2	Tall	4.6	382.47	98.70	16.75	4,308,424	245,350
RSCH68	Nam Hom #2	G5	Dwarf	7.7	350.06	98.73	15.33	2,074,451	118,256
RSCH69	Nam Hom #3	G5	Dwarf	7.9	382.92	98.74	16.78	2,112,589	121,664
RSCH70	Nam Hom #4	G5	Dwarf	8.3	313.20	98.74	13.74	2,041,130	115,977
RSCH71	Nam Hom #5	G5	Dwarf	8.2	289.44	98.76	12.71	1,997,857	113,526
SAL_Cn22_52	Brown sweet #1	G5	Dwarf	7.5	290.16	96.61	12.25	1,012,175	53,552
SAL_Cn22_53	Brown sweet #2	G5	Dwarf	7.5	312.45	96.23	13.12	1,105,773	55,419
SAL_Cn22_81	Maphreaw	Admixture	Tall	7.5	89.47	94.63	5.58	1,342,349	57,232

**Table 2:** List of functional variants significantly associated with the sweetness in 49 coconut accessions.

SNP	Significant Codes	Adjusted R^2^	Ref	ATL	SNP Eff	Gene ID	Annotation
1_135984088	**	0.1264	T	C	missense_variant	Cnu01G011700	Sugar (and other) transporter
1_145882242	**	0.1895	C	T	missense_variant	Cnu01G014570	Fructokinase
1_157345938	***	0.3016	A	G	missense_variant	Cnu01G018720	Sugar (and other) transporter
1_157347379	**	0.1333	T	G	missense_variant	Cnu01G018720	Sugar (and other) transporter
1_157352010	**	0.1184	A	G	missense_variant	Cnu01G018730	Sugar (and other) transporter
1_163329328	***	0.2951	A	G	missense_variant	Cnu01G021210	alpha-1,3/1,6-mannosyltransferase ALG2-like
1_163346328	***	0.2881	A	G	missense_variant	Cnu01G021210	alpha-1,3/1,6-mannosyltransferase ALG2-like
1_168918498	**	0.1522	G	C	missense_variant	Cnu01G023700	Sugar (and other) transporter
2_21952206	**	0.1848	G	A	missense_variant	Cnu02G009280	Sucrose synthase
2_28930455	**	0.1294	A	G	missense_variant	Cnu02G010820	Sucrose nonfermenting 4-like protein
2_28931901	**	0.1823	C	G	missense_variant&splice_region_variant	Cnu02G010820	Sucrose nonfermenting 4-like protein
2_36533852	**	0.1568	G	C	missense_variant	Cnu02G012160	Hexokinase
3_13921395	**	0.1513	T	A	missense_variant	Cnu03G006580	Phosphofructokinase
3_13936725	**	0.1305	T	C	missense_variant	Cnu03G006580	Phosphofructokinase
3_29827288	**	0.1375	G	C,A	missense_variant	Cnu03G011100	Fructokinase
3_35378092	**	0.1374	T	C	missense_variant	Cnu03G012010	Sugar efflux transporter for intercellular exchange
4_120857213	**	0.1501	A	C	missense_variant	Cnu04G012550	Sugar (and other) transporter
4_13014850	**	0.1725	C	T	missense_variant	Cnu04G004950	Hexokinase
4_13016579	**	0.1464	C	T	stop_gained	Cnu04G004950	Hexokinase
4_13016642	**	0.1464	C	A	missense_variant	Cnu04G004950	Hexokinase
4_13016651	**	0.1464	G	A	missense_variant	Cnu04G004950	Hexokinase
4_13017837	**	0.1464	G	C	missense_variant	Cnu04G004950	Hexokinase
4_13017876	***	0.2613	T	C	missense_variant	Cnu04G004950	Hexokinase
4_133364483	**	0.1327	A	T	missense_variant	Cnu04G017640	Hexokinase
4_133364586	***	0.2052	T	TA	frameshift_variant	Cnu04G017640	Hexokinase
5_9583913	**	0.1344	A	G	missense_variant	Cnu05G004150	Sucrose synthase
8_15530732	**	0.1364	C	T	stop_gained	Cnu08G006450	Sugar efflux transporter for intercellular exchange
8_15530840	**	0.1364	G	A	missense_variant	Cnu08G006450	Sugar efflux transporter for intercellular exchange
8_19928242	**	0.1167	C	T	missense_variant	Cnu08G008560	Sugar efflux transporter for intercellular exchange
8_19928244	**	0.136	A	T	missense_variant	Cnu08G008560	Sugar efflux transporter for intercellular exchange
8_19934962	**	0.1222	A	G	missense_variant	Cnu08G008560	Sugar efflux transporter for intercellular exchange
8_19935070	**	0.128	G	C	missense_variant	Cnu08G008560	Sugar efflux transporter for intercellular exchange
8_9181379	**	0.1732	G	T	missense_variant	Cnu08G003810	Phosphofructokinase
8_9182540	***	0.2238	A	T	missense_variant	Cnu08G003810	Phosphofructokinase
8_9913460	**	0.1256	A	G	missense_variant	Cnu08G004000	Hexokinase
9_119160271	***	0.2473	C	A	stop_gained	Cnu09G011120	Sucrose synthase
9_119162558	**	0.1534	A	G	missense_variant	Cnu09G011120	Sucrose synthase
9_121551153	***	0.3985	G	A	missense_variant	Cnu09G012520	Major facilitator superfamily
10_183086020	***	0.3244	T	C	missense_variant	Cnu10G024280	Sugar (and other) transporter
10_192176452	***	0.3358	G	C,A	missense_variant	Cnu10G028040	Sugar efflux transporter for intercellular exchange
10_192177601	***	0.2312	G	A	missense_variant	Cnu10G028040	Sugar efflux transporter for intercellular exchange
10_20363231	**	0.1879	T	C	missense_variant&splice_region_variant	Cnu10G005750	Sucrose nonfermenting 4-like protein
10_57193853	**	0.1463	G	T	missense_variant	Cnu10G013990	beta-fructofuranosidase
11_15951447	***	0.2444	C	T	missense_variant	Cnu11G006980	Hexokinase
11_43908723	**	0.1306	G	T	missense_variant	Cnu11G018990	Sugar (and other) transporter
12_89966688	***	0.2144	C	T	missense_variant	Cnu12G018320	Sugar (and other) transporter
12_89966974	**	0.1693	A	G	missense_variant	Cnu12G018320	Sugar (and other) transporter
12_89967123	***	0.2076	G	A	missense_variant	Cnu12G018320	Sugar (and other) transporter
12_89967124	***	0.2076	C	A	missense_variant	Cnu12G018320	Sugar (and other) transporter
13_65960883	**	0.1461	C	T	missense_variant&splice_region_variant	Cnu13G003960	Sugar (and other) transporter
14_124469111	**	0.1566	A	G	missense_variant	Cnu14G014760	Raffinose synthase or seed imbibition protein Sip1
14_124469119	**	0.1566	A	G	missense_variant	Cnu14G014760	Raffinose synthase or seed imbibition protein Sip1
14_144741125	**	0.1605	A	C	missense_variant	Cnu14G021780	Phosphofructokinase
14_149652548	***	0.2045	G	A	missense_variant	Cnu14G024290	Sugar (and other) transporter
14_149652623	***	0.2593	T	A	missense_variant	Cnu14G024290	Sugar (and other) transporter
14_161558915	***	0.2522	A	G	missense_variant	Cnu14G029310	Sucrose nonfermenting 4-like protein
14_7085864	**	0.12	T	G	missense_variant	Cnu14G002810	Sugar efflux transporter for intercellular exchange

** *p* < 0.01; *** *p* < 0.001.

## Data Availability

The data presented in this study are available on request from the corresponding author. The SNP dataset presented in this study can be found in Zenodo repository (https://doi.org/10.5281/zenodo.13352204). The raw sequencing data generated in this study have been deposited with links to BioProject accession number PRJNA1153526 in the NCBI BioProject database (https://www.ncbi.nlm.nih.gov/bioproject/ (accessed on 1 June 2024)).

## Supplementary Materials

The following supporting information can be downloaded at: https://www.mdpi.com/article/10.3390/plants13182548/s1, Table S1: The CDS sequence of Cnu09G011120 (sucrose synthase) shows a nucleotide change causing a premature stop codon; Table S2: List of genes involved in sucrose metabolism and sugar transport; Table S3: List of functional variants in the genes involved in sugar metabolism and transport tested in 49 coconut accessions.
